# Anesthetic Management for Giant Abdominal Paraganglioma Resection in a Patient After Extracorporeal Membrane Oxygenation: A Case Report

**DOI:** 10.7759/cureus.97649

**Published:** 2025-11-24

**Authors:** Ying Gao, Xu Zhou, Ouyue Zhong, Wei Wu

**Affiliations:** 1 Department of Anesthesiology, The General Hospital of Western Theater Command, Chengdu, CHN

**Keywords:** case report, catecholamine crisis, extracorporeal membrane oxygenation, general anesthesia, paraganglioma

## Abstract

Paraganglioma (PGL) is a rare neuroendocrine tumor that poses significant challenges to anesthetic management during surgical resection. In rare instances, PGL can precipitate a catecholamine crisis, potentially leading to heart failure, intracranial hemorrhage, renal failure, arrhythmias, pulmonary edema, and multisystem organ failure. We report a case of a 53-year-old male with a history of poorly controlled hypertension who was admitted for chest pain and initially diagnosed with non-ST-elevation myocardial infarction (NSTEMI). Coronary angiography revealed no abnormalities. Subsequently, the patient experienced cardiac arrest. Despite aggressive resuscitation, persistent hypoxemia necessitated extracorporeal membrane oxygenation (ECMO) support. Abdominal CT revealed a large retroperitoneal mass suggestive of PGL. Following active resuscitation, the patient was successfully weaned from ECMO. After thorough preoperative optimization and multidisciplinary collaborative surgery with precise anesthetic management, the tumor was successfully resected. This case demonstrates that for patients with PGL experiencing heart failure secondary to catecholamine crisis, tumor resection under general anesthesia can be safely performed after thorough preoperative preparation. Furthermore, this rare case highlights the need for clinicians to consider the possibility of a PGL-induced catecholamine crisis in patients admitted with chest pain, acute myocardial infarction, or acute heart failure.

## Introduction

As a rare neuroendocrine neoplasm of neural crest origin, paraganglioma (PGL) arises in the sympathetic/parasympathetic ganglia. Parasympathetic PGLs predominantly occur in the head and neck region and are typically nonsecreting. In contrast, sympathetic PGLs may develop in multiple anatomical sites, including the thorax, retroperitoneum, and pelvis, and frequently secrete catecholamines such as epinephrine, norepinephrine, and dopamine [1]. In these patients, hypertension is the most common clinical symptom, with some patients potentially presenting with the typical triad of headaches, palpitations, and excessive sweating [2]. PGL secretes catecholamines continuously and abundantly, directly activating adrenalin receptors and inducing tissue damage and cardiac muscle cell fibrosis, resulting in catecholamine-induced cardiomyopathy. Some patients present with hypercalcemia, hematuria, diabetes, or Cushing’s syndrome [3]. Retroperitoneal PGLs account for approximately 10% of sympathetic PGLs, with malignancy rates potentially reaching up to 50% [2]. 

## Case presentation

**Figure 1:**
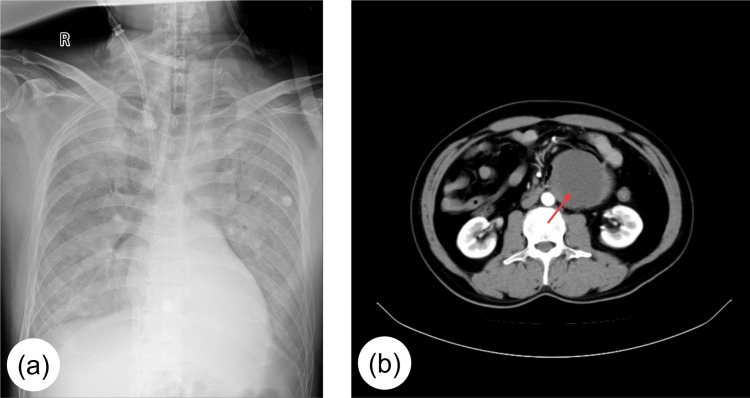
Imaging findings a: chest radiograph during ECMO support; b: abdominal CT scan showing the maximum axial diameter of the tumor (arrow). ECMO: extracorporeal membrane oxygenation

A 53-year-old male patient with poorly controlled hypertension was admitted with severe chest pain for seven hours, accompanied by sweating, chest tightness, nausea, and vomiting. The symptomatology began following minor abdominal trauma. Examination at an external hospital indicated acute non-ST-elevation myocardial infarction (NSTEMI). The patient was transferred to our hospital and admitted to the cardiology department for "heart failure." Coronary angiography revealed no stenosis. During the procedure, the patient developed atrial tachycardia (120-150 bpm), systolic hypotension (80 mmHg), and dyspnea, prompting transfer to the intensive care unit (ICU). The patient underwent tracheal intubation for mechanical ventilation and was administered vasoactive drugs because of severe systolic blood pressure fluctuation (range: 40-200 mmHg). Abdominal CT suggested a possible abdominal tumor, and the urology department recommended catecholamine testing after consultation. On day 2, the patient underwent bedside cardiopulmonary resuscitation and electrical defibrillation for ventricular fibrillation. A diagnosis of acute heart failure and pulmonary edema, along with refractory hypoxemia requiring extracorporeal membrane oxygenation (ECMO) cannulation, was made (Figure 1a). Laboratory results revealed the following plasma catecholamine levels: epinephrine (65517.3 pmol/L), norepinephrine (>235898 pmol/L), and dopamine (1136.7 pmol/L) (Table 1). Phentolamine was administered to control blood pressure. After hemodynamic stabilization, ECMO was discontinued on day 4, and the tracheal tube was removed on day 6. The patient was transferred to the endocrinology department for blood pressure management. Two weeks later, retroperitoneal PGL was considered, and elective resection was planned.

**Table 1:** Laboratory findings and reference values

Test	Patient Value	Normal Range	Unit
Epinephrine	65517.3	Supine Position: ≤605.4; Standing Position: ≤769.0	pmol/L
Norepinephrine	>235898	Supine Position: 414.0-4435.5; Standing Position: 1182.8-10054.0	pmol/L
Dopamine	1136.7	Supine Position: ≤195.7; Standing Position: ≤195.7	pmol/L

The patient had a history of hypertension but did not take antihypertensive drugs regularly. Preoperative management included prazosin hydrochloride administration, fluid expansion, and creatine phosphate sodium for myocardial support. Blood pressure stabilized at 110-130 mmHg. Abdominal CT revealed an 86 × 68 × 70 mm solid-cystic retroperitoneal mass in the left mid-abdomen, adherent to the aorta and bowel (Figure 1b). The electrocardiogram showed a normal sinus rhythm (70 bpm). Echocardiography revealed left atrial enlargement, mild left ventricular hypertrophy, a patent foramen ovale (4-mm shunt), and a left ventricular ejection fraction of 61%. The biomarkers, brain natriuretic peptide and troponin I, were at 163.64 pg/mL and 0.075 μg/L, respectively; routine blood tests showed normal liver and kidney function and normal coagulation. The surgeon planned to perform a "retroperitoneal lesion resection" under general anesthesia.

**Figure 2:**
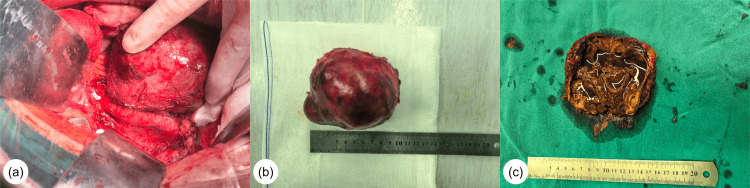
Intraoperative findings a: intraoperative photograph demonstrating the resected tumor; b: en bloc removal of the tumor specimen; c: cross-section of the tumor revealing extensive hemorrhage and necrosis.

After routine fasting and fluid restriction, peripheral venous access was established in the operating room. The preoperative observations were as follows: noninvasive blood pressure, 95/61 mmHg; heart rate, 64 bpm; peripheral oxygen saturation (SpO₂), 98%; bispectral index (BIS), 98. Invasive arterial monitoring and blood gas analysis were also performed. Creatine phosphate sodium (2 g) was administered intravenously before induction. We ensured the availability of essential emergency medications, including nicardipine, phentolamine, esmolol, nitroglycerin, norepinephrine, and epinephrine. Anesthesia was induced with 60 mg of propofol, 6 mg of remimazolam, 50 µg of sufentanil, 14 mg of cisatracurium, and 10 mg of dexamethasone to achieve optimal intubating conditions. The airway was visualized via video laryngoscopy and secured using a 7.0-mm endotracheal tube. Left internal jugular vein catheterization was performed to monitor the central venous pressure. Anesthesia was further maintained using a combination of remifentanil (0.1-0.2 μg/kg/min), dexmedetomidine (24 μg/h), and sevoflurane (2%) to maintain the BIS value within the 40-60 range. Additional cisatracurium (4 mg) and sufentanil (20 μg) were administered before incision. During tumor dissection, an infusion of phentolamine (20-50 mg/h) and esmolol (200-400 mg/h) was initiated, supplemented with intermittent phentolamine boluses. Blood pressure fluctuated between 110 and 170 mmHg before the tumor resection. After four hours, the tumor was successfully removed by the urological, gastrointestinal, and vascular teams. Norepinephrine boluses (4 μg/mL) and infusion (0.05-0.25 μg/kg/min) stabilized the blood pressure at 80-130 mmHg. The operation lasted for five hours; intermittent boluses of cisatracurium (20 mg) and sufentanil (30 μg) were administered. The total fluid infusion volume during the operation was 4,100 mL (including 1,000 mL of hydroxyethyl starch, 2,500 mL of lactated Ringer's solution, and 600 mL of sodium chloride normal saline), three units of red blood cell suspension, and 310 mL of plasma. The surgical bleeding was 400 mL, and the urine output was 1,000 mL. Five minutes after the surgery, the patient regained consciousness, and the tracheal tube was successfully removed. The patient was transferred to the ICU for further observation and then to the urology ward on postoperative day 2. Pathology confirmed a 110 × 70 × 45 mm retroperitoneal PGL with extensive hemorrhage and necrosis. The patient was discharged from the hospital on postoperative day 11 (Figure 2).

## Discussion

Exposure to mechanical forces (e.g., compression or physical stress) may trigger abrupt catecholamine secretion from PGLs. This cascade can precipitate a catecholamine crisis, clinically manifesting as acute pulmonary edema with cardiovascular events, including myocardial infarction, acute heart failure, or other life-threatening complications [4]. The present case involved a patient who was admitted with an acute myocardial infarction and heart failure. Clinical history revealed recent blunt abdominal trauma, likely inducing a catecholamine surge into the circulation. Following admission, the patient received ECMO support due to heart failure and pulmonary edema with refractory hypoxemia. Myocardial injury and impaired pulmonary function complicated perioperative management. Consequently, preoperative optimization lasting approximately two weeks was implemented. Preoperative preparation for PGL resection involves α-adrenergic blockade for vasodilation. If tachycardia or angina develops, β-blockers may be added, but only after adequate α-blockade has been established to prevent hypertensive crises. Additional measures, including aggressive oral hydration and intravenous fluid expansion, reduce the incidence of intra-and postoperative hypotension [5]. During the preparatory phase, the patient received prazosin hydrochloride for vasodilation, creatine phosphate sodium for myocardial support, and substantial volume resuscitation. After the normalization of myocardial injury biomarkers, restoration of pulmonary function, and stable blood pressure control, the patient was scheduled for elective resection. Although small, noninvasive retroperitoneal PGL may be amenable to laparoscopic excision, the large tumor size and dense adhesions to the bowel and abdominal aorta in this case required an open surgical approach [6]. Anesthetic management during PGL resection presents significant challenges owing to profound intraoperative hemodynamic instability, including hypertensive and hypotensive crises, coupled with a high risk of postoperative complications. Therefore, meticulous selection of anesthetic techniques and pharmacological agents is critically important.

PGL resection is typically performed under general anesthesia. Sevoflurane is an acceptable inhalation agent; however, desflurane should be avoided because it may induce hypertension, tachycardia, and airway irritation, which can exacerbate hemodynamic instability in patients with PGL. Propofol is recommended for intravenous sedation, and etomidate may be used for induction in hemodynamically unstable patients. Analgesia is commonly achieved with opioids such as fentanyl, remifentanil, or sufentanil. Neuromuscular-blocking agents, such as vecuronium, rocuronium, or cisatracurium, that increase sympathetic tone or stimulate catecholamine/histamine release (e.g., esketamine, morphine, and pancuronium) are contraindicated in PGL surgery [7]. BIS monitoring enables the real-time assessment of anesthetic depth. An adequate depth suppresses adrenergic and cardiovascular responses. In the present case, sufentanil and remifentanil effectively attenuated surgical stress and facilitated rapid emergence without opioid-related side effects. However, the drawback is that we did not use regional anesthesia to alleviate pain or reduce the dosage of opioid drugs. Although epidural-general anesthesia has been reported to mitigate hemodynamic fluctuations and postoperative complications in pheochromocytoma surgery, a meta-analysis by Yang et al. unexpectedly identified combined epidural-general anesthesia as an independent risk factor for intraoperative and postoperative hypotension compared with general anesthesia alone [8]. The patient did not receive epidural anesthesia. Hemodynamic control was maintained intraoperatively with immediate norepinephrine infusion initiated after the tumor was removed. Owing to thorough preoperative optimization, severe hypotension was avoided, and vasopressor support was discontinued by the end of surgery and the patient was under continuous observation in the ICU.

This patient presented with chest pain and was initially diagnosed with NSTEMI at an external hospital, and coronary angiography at our institution revealed no abnormalities. Takotsubo syndrome may have occurred [9]. This type of cardiopathy is also known as apical ballooning syndrome or stress-induced cardiomyopathy and exhibits clinical features mimicking those of acute coronary syndrome. Typically, left ventricular systolic and diastolic dysfunctions with regional wall motion abnormalities extend beyond the distribution of a single epicardial coronary artery [10]. Although coronary spasms and microvascular ischemia have historically been implicated in their pathogenesis, current evidence has indicated that sympathetic nervous system overstimulation, secondary to psychological or physical stressors, with catecholamine excess as a key pathophysiological mechanism, plays a central role. Inotropic support may be required during heart failure episodes. For patients with Takotsubo cardiomyopathy that progresses to cardiogenic shock, ECMO is an effective therapeutic intervention [11]. Systolic dysfunction and regional myocardial kinetic abnormalities are transient phenomena that typically resolve within days or weeks [12]. This case highlights the importance of considering catecholamine-induced Takotsubo syndrome secondary to PGL as a potential diagnosis in patients presenting with chest pain, acute myocardial infarction, or acute heart failure.

Performing elective surgery in patients treated for acute heart failure poses significant challenges to anesthetic management. This necessitates the precise titration of anesthetic agents, meticulous fluid management, avoidance of profound hemodynamic fluctuations, and prevention of myocardial injury and recurrent acute heart failure. During PGL resection, significant hemodynamic fluctuations occurred, and a large volume of fluid was administered. In addition, standard intraoperative (invasive arterial and central venous pressures) and enhanced cardiac function monitoring are essential. This can be achieved by using modalities such as transesophageal echocardiography (TEE) and pulse-contour cardiac output (PICCO) monitoring [13]. A limitation of this case was the absence of TEE or PICCO, precluding real-time visualization of cardiac motion and assessment of ejection fraction.

## Conclusions

For patients with PGL admitted emergently, thorough preoperative optimization following initial stabilization is crucial. This includes intravascular volume expansion to enhance the intraoperative hemodynamic stability, thereby reducing the risk of cardiovascular and cerebrovascular complications and improving patient outcomes. PGL resection is typically performed under general anesthesia. Preparation of vasoactive drugs, selection of appropriate anesthetic agents, and monitoring of anesthetic depth are essential. Peri-anesthetic management demands titrated precision for surgical procedures in post-ECMO patients. Beyond standard invasive arterial pressure and central venous pressure monitoring, TEE and PICCO monitoring should also be considered when assessing intravascular volume status and cardiac function.
